# The Influence of Saudi Board of Emergency Medicine Residency Educators on Residents’ Academic and Clinical Performance in Riyadh, Saudi Arabia

**DOI:** 10.7759/cureus.54316

**Published:** 2024-02-16

**Authors:** Bader A Alghamdi, Abdulaziz I Alhassan

**Affiliations:** 1 Department of Emergency Medicine, Prince Sultan Military Medical City, Riyadh, SAU; 2 Department of Medical Education, College of Medicine, King Saud bin Abdulaziz University for Health Sciences, Riyadh, SAU; 3 Department of Research, King Abdullah International Medical Research Center, Riyadh, SAU

**Keywords:** academic performance, clinical performance, residency program, residency training program, emergency medicine

## Abstract

Introduction: The Saudi Board of Emergency Medicine (SBEM) is required to assign educators including program directors to supervise their residents. These educators may impact the residents’ academic and clinical performances. After thorough review, we noticed that the current literature lacks information about the direct influence of emergency medicine educators on their residents' academic and clinical performances. The main purpose of this study is to assess the residents’ confidence level, and to measure the program directors’ satisfaction of the residents’ performances during their SBEM training years in Riyadh hospitals.

Methods: This concurrent mixed-methods study was conducted in nine training hospitals in Riyadh, Saudi Arabia, during the 2021-2022 academic year. For the quantitative aspect, a cross-sectional survey was used, based on a questionnaire administered to postgraduate year (PGY)-2 to PGY-4 SBEM residents (n=120) using a nonprobability convenient sampling technique. The survey aimed to assess residents' confidence in their academic and clinical performance, influenced by their educators. A Likert scale with a total of 25 items, covering the seven roles of the CANMEDs framework, was employed. This assessment utilized a published tool called the In-Training Evaluation Report. For the qualitative aspect, program directors' perspectives were gathered through one-on-one unstructured interviews (n=9), guided by theoretical saturation. A purposive sampling technique was employed to select program directors. The interview tool collected demographic data, including gender, years of experience, and training hospital, and included 17 open-ended questions to explore program directors' opinions.

Results: The result of mixed methods showed that both quantitative and qualitative data sets were divergent with all independent variables (resident’s age, gender, and training level) with the exception of the training hospital which was convergent. Thus, the residents’ confidence toward their academic and clinical performances induced by their institutional educators was high. However, the program directors indicated changes that related to residents’ training level.

Conclusion: The residents’ confidence toward their academic and clinical performance induced by their institutional educators was high. The educators had a great deal of influence over the academic and clinical performance of the residents. However, the program directors thought there were some issues with the performance of the residents. Most of the program directors also believed that several influential factors that may have affected the residents’ overall performance include stress and receipt of constructive feedback.

## Introduction

The Saudi Commission for Health Specialties (SCFHS) requires residency training programs to assign program directors (PDs) and educators to supervise, guide and assess residents. The goal of this process is to improve the knowledge and skills of residents in a variety of areas within clinical and academic performance. Since focus is mainly on residents improving their clinical performance, these residency training programs are designed to improve many other areas of knowledge including both clinical and academic performance. By working with program directors and educators, the residents can improve not only how they provide care, but also their skills as leaders, collaborators, and even scholars. Therefore, establishing the resident’s competence as a clinician in different educational and training hospitals in the Kingdom of Saudi Arabia (KSA) [1].

As the emergency medicine (EM) residency program is a part of SCFHS disciplines, different PDs and educators were assigned in many hospitals, who may have an impact on the residents’ academic and clinical performances during the four-year residency program till they graduate and certify [1]. The ability to work with different program directors and educators may be beneficial to residents so that they can gain knowledge and insights from a variety of people. In this way, residency training better prepares residents for the situations, conditions, and skills they might need once they are certified.

Typically, each resident in the program undergoes distinct and evolving experiences, with their academic and clinical performance evolving as they progress from one year to the next. As a result, there might be a possibility that the personal characteristics of the residents will affect the training received during the residency program and impact the overall performance and confidence of the residents. There are several factors in the residents’ lives that could influence this change in performance [2]. The demographic factors of the residents may be important and significant in influencing how the residents perceive their performance as part of their residency training. However, more research is needed to understand how personal demographic factors may impact how the residents perceive their clinical and academic performance as part of the residency training.

It is also important to understand that the PDs and other educators could play a fundamental role in the residents’ performances, ranging from considering those educators as role models, to continuously assessing and delivering sufficient feedback. Several studies have been conducted regarding residents’ performances, but none addressed the direct effect of educators on their residents’ performances [2-5]. The lack of studies about the direct effect of educators on the performance of residents is a major gap within the academic literature. The amount of time that educators spend with residents makes it worthwhile to examine if there is a direct effect between educators and residents’ performances. The importance of residency programs on the training of residents and their future certifications warrants investigation into the effect, whether direct or indirect, of educators on their residents during training.

One study was conducted by Mittelman et al. who found that some factors (e.g. academic challenges, professionalism) could affect the resident’s completion of the EM program and consequently change their specialty [6]. Whereas Albano et al. established that well-constructed feedback will affect the resident’s performance [7]. In this way, program directors and educators need to be aware of how they provide feedback so that the residents are able to use the feedback to improve their performance. Otherwise, residents may not complete the emergency medicine training and instead switch to other specialties.

Alsalamah and Almadani found that the Saudi Board of Emergency Medicine (SBEM) residents in Riyadh city are generally satisfied with their program but the constructive feedback is insufficient and needs to improve [8]. According to Yao, the medical knowledge and clinical skills are important factors for clinical competency among internal medicine residents, and the residency educators should work on remediating any problem associated with these factors during the residency program [5]. In this regard, residents need to understand how they are performing and receive feedback that can be used to improve in areas where they may not be performing as needed. Otherwise, the residents may not realize that they need to improve their performance or may not understand in which specific areas they need to improve their performance. If this is true, then this may be an important aspect of the residency program and residency education that needs to be improved in order to improve the overall training that residents receive.

In addition, Whitley et al. concluded that EM residency program supervisors should be aware of the effects of stress on the residents which may have an impact on their behaviors and performances [3]. The stress that residents feel as they complete an important part of their training may affect how they receive the information that is provided, as well as their confidence with regard to the duties and roles they are expected to carry out. However, more stress may not be an entirely negative aspect of the residency training program.

LeBlanc and Bandiera conclude that emergency medicine junior residents performed better under stress, while the performance of more experienced residents was not affected by the stress condition [9]. A study has shown that increasing the level of anxiety among EM residents can lead to greater confidence in managing common clinical conditions, and competence in performing emergency procedures as compared to other specialties. Moreover, the PDs should be aware of and remediate the effects of stress on the residents' performances [10]. Rather than trying to reduce or eliminate stress for the residents, it might be better for the program directors and educators to utilize stress as a tool to help residents adjust to the clinical conditions they will face in emergency medicine throughout their careers.

After an extensive search in the literature review, no study was found addressing the direct influence of EM educators on their residents’ academic and clinical performances [2-5]. The lack of research about the influence of EM educators on their residents is problematic given the importance of these individuals in training EM residents. While it may be assumed that program directors and educators have a great impact on the training and confidence of residents, it is not known whether this is true in practice. Furthermore, it is not known how residents perceive their own performance or how program directors perceive the performance of residents. Therefore, the main purpose of this study is to assess the residents’ confidence level and to measure the program directors’ satisfaction about the residents’ performance during their training years within the SBEM program in Riyadh hospitals.

In addition, using mixed methods methodology is recommended in medical education because it can increase the integrity and applicability of findings [11,12]. For this reason, mixed methods methodology is used in this study. By using a mixed methods methodology, it is possible to examine how the residents perceive their own academic and clinical confidence. At the same time, the perceptions of the program directors regarding the performance of the residents can be examined. With the combination of quantitative and qualitative data from the residents and program directors, it will be possible to gain a higher level of understanding about the clinical and academic performance of the residents and why specific performance or knowledge issues arise within the data. For example, if the residents indicate that they perceive themselves to have lower performance in a particular clinical or academic area, the qualitative data collected from the program directors may be useful in understanding the reason for these perceptions.

Thus, the aim of this study is to assess the residents’ confidence level and to measure the program directors’ satisfaction of the residents’ performance during their training years within the SBEM program in Riyadh hospitals. In the quantitative method, the objective is to measure the residents’ confidence in the effect of their institutional educators on their academic and clinical performance during their SBEM residency program in Riyadh hospitals. While in the qualitative method, the objective is to explore the residents’ academic and clinical performance in the SBEM residency program through the program directors’ perception. Lastly, the objective of the mixed methods method is to compare the residents’ confidence with their program directors’ perception of the residents’ academic and clinical performance in SBEM in Riyadh hospitals.

## Materials and methods

Study area/setting

Both quantitative and qualitative methods were conducted in all nine EM training hospitals in Riyadh city during the residents’ educational day where they were all gathered with their PDs in a lecture hall in each center. The nine residency training hospitals are Prince Sultan Military Medical City (PSMMC), King Abdulaziz Medical City (KAMC), King Fahd Medical City (KFMC), King Saud Medical City (KSMC), King Faisal Specialist Hospital and Research Center (KFSHRC), Prince Mohamed Bin Abdulaziz Hospital (PMAH), Security Force Hospital (SFH), King Khalid University Hospital (KKUH) and, Dr. Sulaiman Alhabib Hospital (HMG). 

Study design

The quantitative study employed a cross-sectional design, which involved administering a survey to examine the residents' characteristics. It utilized a Likert scale to assess the confidence level of the residents in their academic and clinical performance progress, which was influenced by their educators. Data was collected electronically within the survey. The qualitative study was a one-to-one respondent unstructured individual interview. Each program director was requested to answer open-ended questions stating their opinions on their residents academic and clinical performance progress as well as the possible influential factors that might affect their performance. The final data was then collected and analyzed.

Study population

In the quantitative method, all residents (postgraduate year (PGY) 2-4) who actively joined the SBEM program in all residency training hospitals in Riyadh City, Saudi Arabia were included. Exclusion criteria included any training hospitals who did not have senior residents, all PGY-1 SBEM residents, and all SBEM residents outside Riyadh city. The qualitative method, included SBEM program directors in all residency training hospitals in Riyadh City, Saudi Arabia. It excluded any SBEM program directors who did not have SBEM residents PGY-2 to PGY-4.

Quantitative method

The sample size for this study was determined to be 120 residents to achieve a confidence level of 95% with a margin of error of 7%. We employed a nonprobability convenient sampling technique. The questionnaire used in this study was divided into two parts. The first part collected demographic data, including the resident's age, gender, residency level, and training hospital. The second part aimed to assess the residents' confidence in their academic and clinical performance, as influenced by their educators. This assessment used a Likert scale consisting of a total of 25 items, which covered the seven roles outlined in the CANMEDs framework. We utilized a validated tool called the In-Training Evaluation Report (ITER), which exhibited a high level of internal consistency with a Cronbach's alpha reliability coefficient of 0.97 [13]. The CANMEDs roles assessed in the Likert scale included medical expert, communicator, collaborator, manager, scholar, health advocate, and professional. The Likert scale ranged from 1 to 5, with 1 indicating 'Not confident at all,' 2 indicating 'Slightly confident,' 3 indicating 'Somewhat confident,' 4 indicating 'Fairly confident,' and 5 indicating 'Completely confident.' Both parts of the questionnaire were categorized as variables. The main independent variables considered in this questionnaire were the CANMEDs framework, resident's age, resident's gender, training level, and the training hospital. The dependent variable was the resident's confidence.

Qualitative method

The sample consisted of nine program directors, selected through purposive sampling guided by theoretical saturation. Data collection continued until data saturation was achieved, at which point the interviews were concluded. These semi-structured interviews were conducted in an audio-recorded format, involving open-ended questions posed to each program director regarding their opinions on their residents' academic and clinical performance, as well as potential factors influencing residents' performance. Each interview had an average duration of 40-60 minutes per participant. Prior to the interviews, all participants were asked to sign a consent form. The researcher also provided a comprehensive explanation of the study's purpose, emphasized the value of their input, and clarified that participation was voluntary, with the right to withdraw from the interview at any time. During the interviews, the researcher adhered to an interview guide that included open-ended questions aligned with the study objectives. Demographic data such as gender and years of experience were collected alongside the interviews. To ensure question clarity, the interview guide was piloted with three consultants from the emergency medicine residency program. The interview guide was structured around the roles defined in the CANMEDs framework. All interviews were recorded for the purpose of ensuring data collection and analysis quality.

Ethical considerations

All residents and program directors in this study are voluntary participants, and each one was allowed to withdraw from the study at any time. Each questionnaire included a consent form with a cover letter explaining the purpose of the study and reassuring the residents and the program directors about the confidentiality of their scoring. Ethics approval was obtained from the Institutional Review Board of the King Abdullah International Medical Research Center, National Guard Health Affairs, Riyadh, Saudi Arabia (IRB/2457/22).

Statistical analysis

Quantitative Method

Statistical Package for Social Sciences (SPSS) version 25.0 (IBM Corp., Armonk, NY, USA) was used for statistical analysis. Descriptive (frequency, percentage, mean, and standard deviation) and inferential analysis (Fisher’s exact test) were used in this study. The overall confidence of the residents was calculated by averaging a total of 25 items. Then, the confidence level was categorized into three levels: Low (overall mean score <2), Medium (overall mean score 2-3), and High (overall mean score >3). Data entry and analysis were carried out using the statistical program SPSS. All categorical variables were presented as frequencies (n), percentages (%), mean, and standard deviations. Fisher’s exact test was used to assess the association between the level of confidence and the demographic data of the residents.

Qualitative Method

All the recorded interviews were transcribed verbatim. Then, the transcriptions were entered onto a qualitative data management software (NVivo 12). The data was analyzed using thematic analysis approach in which the texts were coded, and then further grouped into similar codes together. The similar codes were categorized into subthemes that formulate the major themes of the study. For validity and credibility, the data was checked by two members [14].

## Results

Quantitative

The purpose of this study is to describe the results of the study, which designed to measure the effectiveness of Saudi board of emergency medicine program educators on the residents’ performance in Riyadh hospitals through In-Training Evaluation Report (ITER), Riyadh, Saudi Arabia. Statistical package for the social sciences (IBM-SPSS®) (version 25.0) was used for statistical analysis. Descriptive (frequency, percentage, mean, and standard deviation) and inferential analysis (Fisher’s exact test) were used in this study.

Characteristics of participants

To determine the characteristics of participants, frequencies and percentage were used, as shown in Table 1 as follows:

**Table 1:** The distribution of the overall study sample according to their demographic characteristics (n=120) * The data has been represented as numbers and percentage PGY: postgraduate year

Selected Characteristics		Frequency	Percent
Gender	Male	77	64.2
	Female	43	35.8
Age	25-29	105	87.5
	30-34	14	11.7
	35-39	1	.8
I am a Saudi board of emergency medicine resident	PGY-2	41	34.2
	PGY-3	33	27.5
	PGY-4	46	38.3
My training hospital is	Prince Sultan Military Medical City	24	20
	King Abdulaziz Medical City	29	24.2
	King Saud Medical City	20	16.7
	Security Force Hospital	4	3.3
	King Faisal Specialized Hospital	9	7.5
	King Khalid University Hospital	5	4.2
	Dr. Sulaiman Alhabib Hospital	1	0.8
	King Fahd Medical City	22	18.3
	Prince Mohamed bin Abdulaziz Hospital	6	5

**Figure 1:**
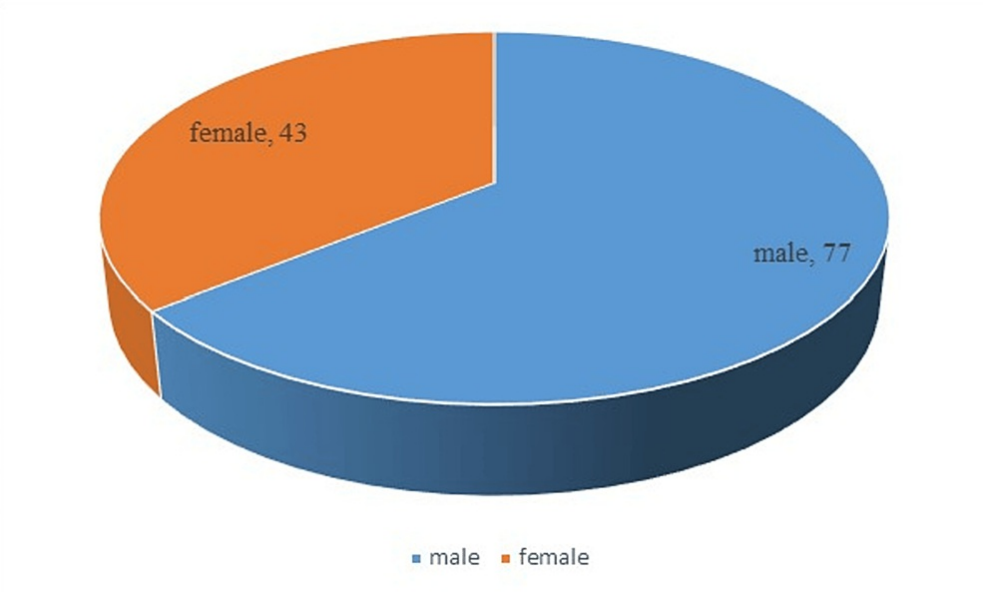
Distribution of respondents according to gender

**Figure 2:**
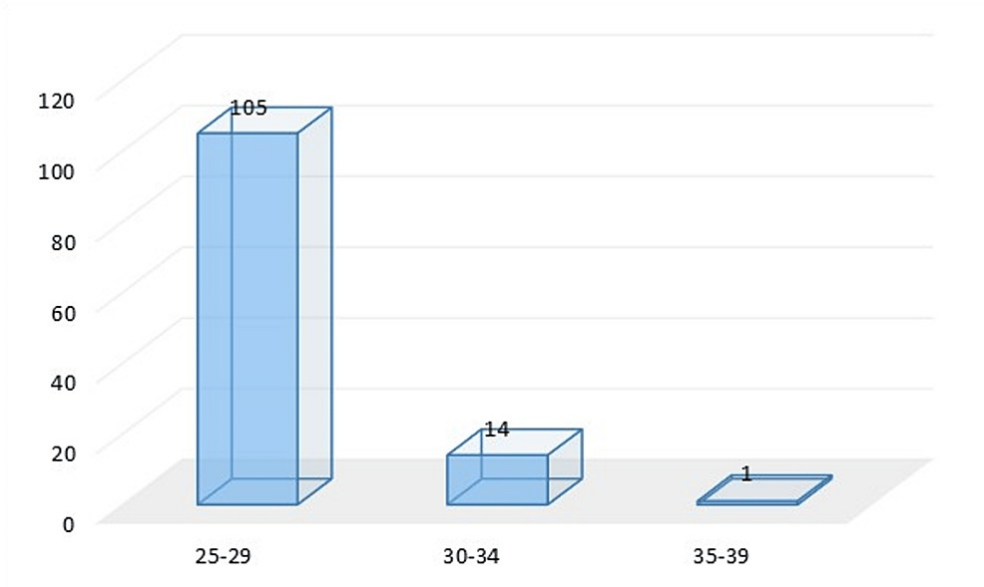
Distribution of respondents according to age

**Figure 3:**
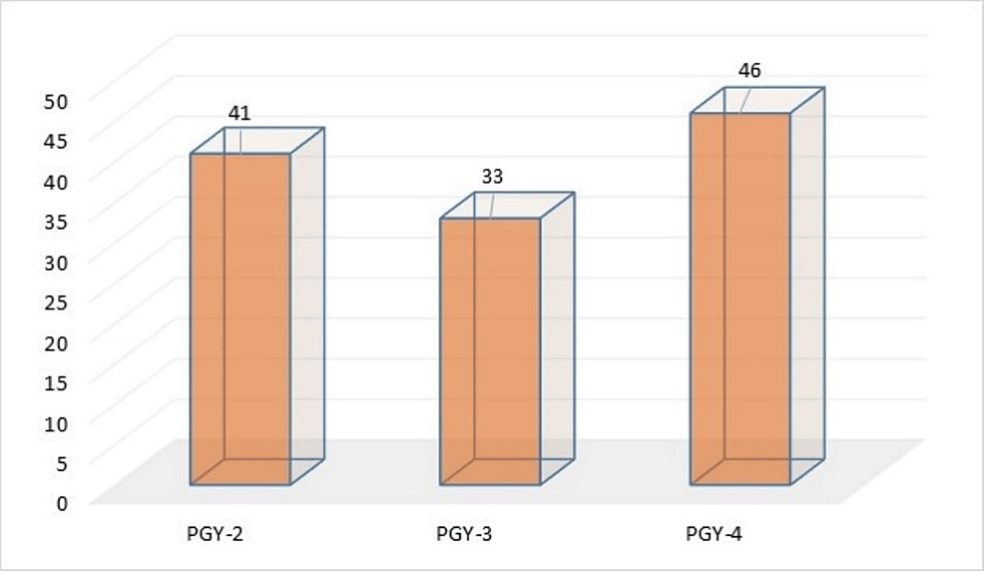
Distribution of respondents according to training level

**Figure 4:**
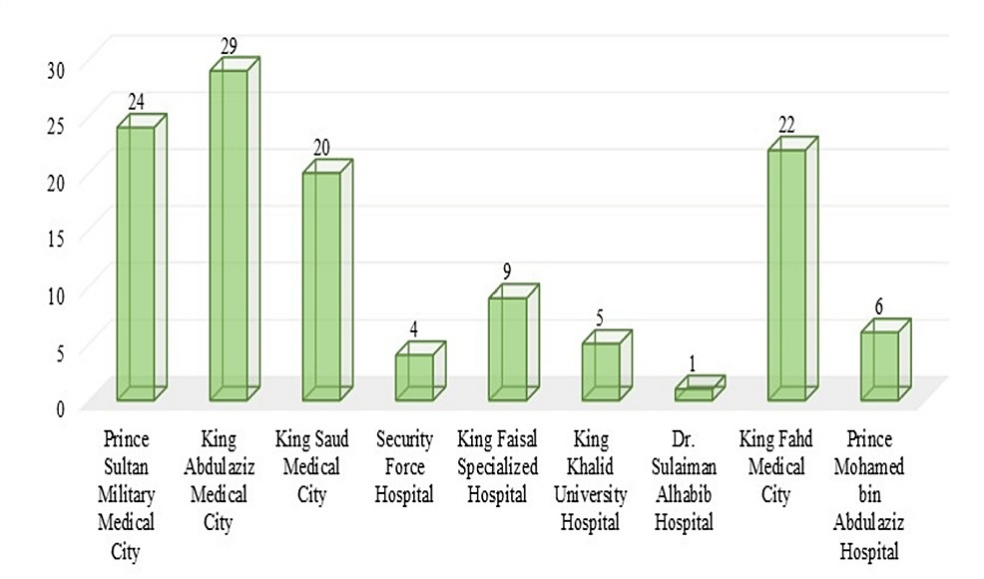
Distribution of respondents according to training hospital

Results listed in Table 1 and Figures 1, 2, 3, 4 showed that the majority of respondents were males 77 (64.2%), while 43 (35.8%) were females, with regard to the age variable, the vast majority of respondents 105 (87.5%) were aged between 25-29 years old, while 12.5% were aged between 30-39 years old.

With regard to the training level, 46 (38.3%) residents were at PGY-4, while 33 (27.5%) respondents were at PGY-3. With regard to the training hospital, there were 29 (24.2%) respondents in King Abdulaziz Medical City, while one (0.8%) respondent was in Dr. Sulaiman Alhabib Hospital.

Respondents' confidence toward their academic and clinical performance

To determine the respondent’s confidence toward their academic and clinical performance, mean and standard deviation of the individuals' responses were calculated as follows:

Medical Expert

To determine the respondent’s confidence toward their academic and clinical performance related to Medical Expert, mean and standard deviation of the individuals' responses were calculated as follows in Table 2.

**Table 2:** The respondent’s confidence toward their academic and clinical performance related to Medical Expert (n=120) * The data has been represented as Mean±SD

N	Items	Mean	SD	ranking
1	I am comprehensive, accurate and concise with all relevant details about the history and physical examination.	4.24	0.69	1
2	I use the diagnostic tests in a cost-effective manner and understands limitations and predictive value.	3.63	0.78	8
3	I am able to formulate an appropriate differential diagnosis.	4.12	0.75	2
4	I am able to analyze, integrate, and formulate effective management strategies.	3.86	0.75	5
5	I have a broad clinical and basic knowledge of a wide variety of medical problems and develops a plan of secondary prevention.	3.62	0.82	9
6	I am able to identify and respond appropriately to urgent cases.	4.09	0.83	3
7	I am aware of the role of evidence in clinical decision-making.	3.81	1.03	7
8	I am able to apply relevant information in problem-solving.	3.83	0.79	6
9	I am able to demonstrate knowledge of medications used, mechanisms of action, clinically relevant pharmacokinetics, indications, contraindications, and adverse effects.	3.17	1.05	10
10	I am able to perform diagnostic and therapeutic procedures, understand indications, limitations and complications.	3.95	0.84	4
Overall mean		3.83	0.59	-

Table 2 showed the results revealed that respondents exhibited high confidence in their academic and clinical performance in the 'Medical Expert' domain, with a mean score of 3.83 ± 0.59. Notably, item number 1, which assesses the ability to be comprehensive, accurate, and concise in documenting relevant details about history and physical examination, received the highest perception and ranked first with a mean score of 4.24 ± 0.69, indicating 'complete confidence.' Following closely, item number 3, evaluating the capability to formulate appropriate differential diagnoses, received a mean score of 4.12 ± 0.75, signifying 'fairly confident.' In the third position, item number 6, which relates to the ability to identify and respond appropriately to urgent cases, received a mean score of 4.09 ± 0.83, also indicating 'fairly confident.' Conversely, item number 9, assessing the ability to demonstrate knowledge of medications, including mechanisms of action, clinically relevant pharmacokinetics, indications, contraindications, and adverse effects, ranked last with a mean score of 3.17 ± 1.05, signifying 'somewhat confident.

Communicator

To determine the respondent’s confidence toward their academic and clinical performance related to communicator, mean and standard deviation of the individuals' responses were calculated as follows in Table 3.

**Table 3:** The respondent’s confidence toward their academic and clinical performance related to communicator (n=120) * The data has been represented as Mean±SD

N	Items	Mean	SD	ranking
1	I am able to communicate effectively with patients, their families, and HCPs.	4.51	0.70	1
2	I am able to maintain clear, accurate and appropriate records.	4.22	0.80	3
3	I am able to write an organized and legible orders and progress notes.	4.38	0.72	2
4	I am able to document a concise and complete discharge summaries promptly.	4.02	0.98	4
Overall mean		4.28	0.65	-

Table 3 showed that the respondents had very high confidence in their academic and clinical communication skills related to the 'communicator' domain, scoring an average of 4.28 ± 0.65. In this context, item number 1, 'I am able to communicate effectively with patients, their families, and healthcare professionals,' was highly perceived and ranked first with an average score of 4.51 ± 0.70, indicating 'complete confidence.' Following closely, item number 3, which assesses the ability to 'write organized and legible orders and progress notes,' received an average score of 4.38 ± 0.72, also indicating 'complete confidence.' In the third position, item number 2, related to 'maintaining clear, accurate, and appropriate records,' received an average score of 4.22 ± 0.80, again indicating 'complete confidence.' Conversely, item number 4, focusing on 'documenting concise and complete discharge summaries promptly,' ranked last with an average score of 4.02 ± 0.98, signifying 'fairly confident.

Collaborator

To determine the respondent’s confidence toward their academic and clinical performance related to Collaborator, mean and standard deviation of the individuals' responses were calculated as follows in Table 4.

**Table 4:** The respondent’s confidence toward their academic and clinical performance related to communicator (n=120) * The data has been represented as Mean±SD

N	Items	Mean	SD	ranking
1	I am able to work effectively in a team environment with attending, juniors and nursing staff.	4.51	0.66	-
Overall mean		4.51	0.66	-

Table 4 showed that the respondent’s confidence toward their academic and clinical performance related to Collaborator was very high with a mean score of 4.51 ± 0.66. This answers shows that respondents' are able to work effectively in a team with other colleagues including juniors and nursing staff.

Manager

To determine the respondent’s confidence toward their academic and clinical performance related to manger, mean and standard deviation of the individuals' responses were calculated as follows in Table 5.

**Table 5:** The respondent’s confidence toward their academic and clinical performance related to communicator (n=120) * The data has been represented as Mean±SD

N	Items	Mean	SD	ranking
1	I am able to serve in administration and leadership roles as appropriate.	3.71	1.00	2
2	I am able to use the health care resources appropriately and efficiently.	3.83	0.85	1
Overall mean		3.77	0.81	-

The findings showed in Table 5 revealed that respondents expressed high confidence in their academic and clinical performance in the 'manager' dimension, with a mean score of 3.77 ± 0.81. Notably, item number 2, 'I am able to use healthcare resources appropriately and efficiently,' secured the top position with a mean score of 3.83 ± 0.85, indicating 'fairly confident.' Conversely, item number 1, 'I am able to serve in administration and leadership roles as appropriate,' ranked last with a mean score of 3.71 ± 1.0, also indicating 'fairly confident.

Scholar

To determine the respondent’s confidence toward their academic and clinical performance related to scholar, mean and standard deviation of the individuals' responses were calculated as follows in Table 6.

**Table 6:** The respondent’s confidence toward their academic and clinical performance related to scholar (n=120) * The data has been represented as Mean±SD

N	Items	Mean	SD	ranking
1	I attend and contribute to rounds, seminars, and other learning events.	3.82	1.05	3
2	I accept and act on constructive feedback.	4.29	0.88	1
3	I contribute to the education of patients, junior residents, house staff, and students.	4.08	0.86	2
4	I contribute in scientific research.	2.92	1.34	4
Overall mean		3.78	0.76	-

The results presented in Table 6 indicate that respondents expressed high levels of confidence in their academic and clinical performance in the 'scholar' domain, with an average score of 3.78 ± 0.76. Notably, item number (2), 'I accept and act on constructive feedback,' ranked first with an average score of 4.29 ± 0.88, signifying 'complete confidence.' Following closely, item number (3), which evaluates the ability to 'contribute to the education of patients, junior residents, house staff, and students,' received an average score of 4.08 ± 0.86, indicating 'fairly confident.' In the third position, item number (1), related to 'attending and contributing to rounds, seminars, and other learning events,' received an average score of 3.82 ± 1.05, also indicating 'fairly confident.' Meanwhile, item number (4), focusing on 'contributing to scientific research,' ranked last with an average score of 2.92 ± 1.34, signifying 'somewhat confident.

Health Advocate

To determine the respondent’s confidence toward their academic and clinical performance related to health advocate, mean and standard deviation of the individuals' responses were calculated as follows in Table 7.

**Table 7:** The respondent’s confidence toward their academic and clinical performance related to health advocate (n=120) * The data has been represented as Mean±SD

N	Items	Mean	SD	ranking
1	I am able to identify the psychosocial, economic, environmental and biological factors, which influence the health of patients and society.	3.67	0.96	2
2	I offer advocacy on behalf of patients at practice and general population levels.	3.75	0.95	1
Overall mean		3.71	0.90	-

The findings in Table 7 revealed that the respondent’s confidence toward their academic and clinical performance related to health advocate was high with a mean score of 3.71 ± 0.90. Item number (2) “I offer advocacy on behalf of patients at practice and general population levels” ranked first with a mean score of 3.75 ± 0.95 which refers to “fairly confident”, while item number (1) “I am able to identify the psychosocial, economic, environmental and biological factors, which influence the health of patients and society” ranked last with a mean score of 3.67 ± 0.96 which refers to “fairly confident”.

Professional

To determine the respondent’s confidence toward their academic and clinical performance related to Professional, mean and standard deviation of the individuals' responses were calculated as follows in Table 8.

**Table 8:** The respondent’s confidence toward their academic and clinical performance related to Professional (n=120) * The data has been represented as Mean±SD

N	Items	Mean	SD	ranking
1	I deliver the highest quality of care with integrity and compassion, and I recognize limitations and seek advice and consultations when necessary.	4.25	0.76	1
2	I reflect the highest standards of excellence in clinical care and ethical conduct.	4.07	0.87	2
Overall mean		4.16	0.74	-

Table 8 showed that the respondent’s confidence toward their academic and clinical performance related to Professional was very high with a mean score of 4.16 ± 0.74. Item number (1) “I deliver the highest quality of care with integrity and compassion, and I recognize limitations and seek advice and consultations when necessary” ranked first with a mean score of 4.25 ± 0.76 which refers to “completely confident”, while item number (2) “I reflect the highest standards of excellence in clinical care and ethical conduct” ranked last with a mean score of 4.07 ± 0.87 which refers to “fairly confident”.

The previous results showed that, the respondent’s confidence toward their academic and clinical performance were as follows in Table 9.

**Table 9:** The resident’s confidence toward their academic and clinical performance (n=120) * The data has been represented as Mean±SD

N	Dimensions	Mean	SD	ranking
1	Medical expert	3.83	0.59	4
2	Communicator	4.28	0.65	2
3	Collaborator	4.51	0.66	1
4	Manager	3.77	0.81	6
5	Scholar	3.78	0.76	5
6	Health advocate	3.71	0.90	7
7	Professional	4.16	0.74	3
Overall mean		3.93	0.53	-

The results presented in Table 9 indicate that respondents exhibited high levels of confidence in their academic and clinical performance, with an overall mean score of 3.93 ± 0.53. Notably, the 'collaborator' dimension ranked first, achieving an impressive mean score of 4.51 ± 0.66. Following closely, the 'communicator' dimension secured the second position with a mean score of 4.28 ± 0.65. In the third place, the 'professional' dimension received a mean score of 4.16 ± 0.74.

Conversely, the 'medical expert' dimension occupied the fourth rank with a mean score of 3.83 ± 0.59. Subsequently, the 'scholar' dimension followed with a mean score of 3.78 ± 0.76, and the 'health advocate' dimension trailed with a mean score of 3.71 ± 0.90. Lastly, the 'professional' dimension ranked last with a mean score of 4.16 ± 0.74.

The association between the level of confidence and baseline characteristics of the residents

The Association Between the Level of Confidence and Respondent’s Gender

To determine the association between the level of confidence and respondent’s gender, Fisher’s exact test was calculated as follows in Table 10.

**Table 10:** The association between the level of confidence and respondent’s gender (n=120) * The data has been represented as number and percentage

Confidence level		Gender		Total
		Male	Female	
Medium	n	2	3	5
	%	1.7%	2.5%	4.2%
High	n	75	40	115
	%	62.5%	33.3%	95.8%
Total	n	77	43	120
	%	64.2%	35.8%	100.0%
	Fisher’s exact:1.325 P- value: 0.250			

Table 10 indicates that there is no statistically significant correlation between the respondents' level of confidence and their gender. The significance level, with a value of 0.250, is greater than the standard threshold of 0.05, signifying that the lack of significance is not statistically meaningful.

The Association Between the Level of Confidence and Respondent’s Age

To determine the association between the level of confidence and respondent’s age, Fisher’s exact test was calculated as follows in Table 11.

**Table 11:** The association between the level of confidence and respondent’s age (n=120) * The data has been represented as number and percentage

Confidence level		Age			Total
		25-29	30-34	25-39	
Medium	n	4	1	0	5
	%	3.3%	0.8%	0.0%	4.2%
High	n	101	13	1	115
	%	84.2%	10.8%	0.8%	95.8%
Total	n	105	14	1	120
	%	87.5%	11.7%	0.8%	100.0%
	Fisher’s exact: 0.388 P- value: 0.824				

The results presented in Table 11 show that there is no statistically significant correlation between the level of confidence and the respondents' age. The significance level, amounting to 0.824, is greater than the standard threshold of 0.05, indicating that the lack of significance is not statistically meaningful.

The Association Between the Level of Confidence and Respondent’s Training Level

To determine the association between the level of confidence and respondent’s training level, Fisher’s exact test was calculated as follows in Table 12.

**Table 12:** The association between the level of confidence and respondent’s training level (n=120) * The data has been represented as number and percentage

Confidence level		Training Level			Total
		PGY-2	PGY-3	PGY-4	
Medium	n	3	1	1	5
	%	2.5%	0.8%	0.8%	4.2%
High	n	38	32	45	115
	%	31.7%	26.7%	37.5%	95.8%
Total	n	41	33	46	120
	%	34.2%	27.5%	38.3%	100.0%
	Fisher’s exact: 1.583 P- value: 0.453				

In Table 12, it is evident that there is no statistically significant correlation between the level of confidence and the respondents' training level. The significance level, measuring at 0.453, surpasses the conventional threshold of 0.05, signifying that this lack of significance is not statistically significant.

The Association Between the Level of Confidence and Respondent’s Training Hospital

To determine the association between the level of confidence and respondent’s training hospital, Fisher’s exact test was calculated as follows in Table 13.

**Table 13:** The association between the level of confidence and respondent’s training hospital (n=120) * The data has been represented as number and percentage

Training hospital		Confidence Level		Total
		Medium	High	
Prince Sultan Military Medical City	n	3	21	24
	%	2.5%	17.5%	20.0%
King Abdulaziz Medical City	n	1	28	29
	%	0.8%	23.3%	24.2%
King Saud Medical City	n	0	20	20
	%	0.0%	16.7%	16.7%
Security Force Hospital	n	0	4	4
	%	0.0%	3.3%	3.3%
King Faisal Specialized Hospital	n	0	9	9
	%	0.0%	7.5%	7.5%
King Khalid University Hospital	n	0	5	5
	%	0.0%	4.2%	4.2%
Dr. Sulaiman Alhabib Hospital	n	0	1	1
	%	0.0%	0.8%	0.8%
King Fahd Medical City	n	1	21	22
	%	0.8%	17.5%	18.3%
Prince Mohamed bin Abdulaziz Hospital	n	0	6	6
	%	0.0%	5.0%	5.0%
Total	n	5	115	120
	%	4.2%	95.8%	100.0%
	Fisher’s exact: 6.176 P- value: 0.628			

The results presented in Table 13 indicate that there is no statistically significant correlation between the level of confidence and the respondents' training hospital. The significance level, recorded as 0.628, exceeds the standard threshold of 0.05, signifying that this absence of significance is not statistically significant.

Qualitative

The qualitative interview guide contained in the appendix was used to obtain the qualitative data from the program directors. From the interviews, three main themes emerged in this study, Educational Atmosphere, Program Process, and Trainer’s Behaviors in Educational Program. Each theme had its own list of subthemes. Theme 1, Educational Atmosphere, included Active learning, Theoretical and clinical experience, and External motivation. Theme 2, Program Process had Four subthemes: Educational resources, Educational strategies, Leadership and management, and Educational assessment and evaluation. Theme 3, Trainer’s Behaviors in Educational Program consisted of the following subthemes: Resident lifestyle, and Ethics and professionalism.

The responses provided related to the theme of active learning showed that the program directors recognized differences in performance and skills among the residents. For example, one of the program directors stated that “overall in my center, writing medical records is insufficient due to several factors. First, not all residents have the same writing skills.” Another program director explained:

“Some of the residents learned fast and they have a good motor skill compared to others and some residents do have the ability to learn from the first time compared to others where they take time and multiple attempts to master or to reach a specific competency” (4-2-2-1).

In terms of theoretical and clinical experiences, the program directors explained that the residence process is a journey that results in the residents gaining more skills and improving their clinical performance as they progress through the program. One of the program directors stated:

“The residency is like a journey, they start with almost zero experience when they're juniors who still need close guidance and supervision by the faculty and the instructors. Then, they will start to build up their own experiences to prepare them to graduate as a competent physician” (3-2-2-1).

Another program director explained the reason for the variation in the performance of the residents by stating that

“Another reason for the variation in performing emergency procedures is the opportunities of having procedures, the higher number of procedures been done, the better the performance” (4-2-2-1).

With regards to external motivation, the program directors seemed to believe that a combination of factors that included positive academic experiences during the residency program increased the external motivation of the residents. One of the program directors explained that “the factors that affect my residents performances positively are; motivation, encouragement, rewarding, fulfilling their needs.” Another program director stated that “positive factors are mentorship program, well-structured academic activities, and the supportive work environment.”

For the program process theme, the subtheme of educational resources was important because the program directors indicated that they believed that the residents did not use the resources properly, but also explained that there were reasons for the lack of proper use of resources. One of the program directors clarified that residents' overuse of resources stemmed from their concern for patient safety:

“Most of my residents from all levels they do over investigate and have some element of protective medicine by requesting extra lab and image orders to rule out all emergency diagnoses for all patients aiming to be safe doctors. I believe the main reason beyond that is lack of understanding the concept of health care resources and its utilization among my residents” (4-2-2-1).

However, another program director believed that the overuse of resources on the part of the residents was due to a lack of understanding about the cost of resources:

“As my residents work in a governmental hospital, the awareness of the prices is very limited and this is another factor that make them over investigators. Thus, I think if they knew how much is the cost of these resources they're using, it could have changed the practice” (3-2-2-1).

The next subtheme related to program process was educational strategies. One of the program directors explained that simulations were used to handle any gaps in the clinical knowledge of the residents: “If they have any struggles with any procedures, we try to finish up this gap by doing simulation workshops.” However, another program director stated that the process of addressing any clinical gaps in knowledge was much more complex:

“Clinically, having more discussions and brainstorming sessions regarding their patients during the clinical shifts regarding their cases, giving them the confidence they earn during the shifts, giving them the ability to make decisions on their own, giving them leadership positions during the shifts, giving them more responsibility, making them feel that they have earned the right to be there and work with these patients and they earned the trust that is given to them” (5-1-1-1).

In terms of the subtheme of leadership and management, the program directors indicated that they were not satisfied with their leadership skills as related to communication. One of the program directors explained:

“Good communication with other health care providers as well as with patients plays a critical role in team dynamics and patient trust. Nevertheless, not all PGY-2 to PGY-4 communicate well with others, and this needs to be improved” (1-1-1-1).

The fourth subtheme of program process, which was educational assessment and evaluation, showed that some of the program directors perceived that the residents were only engaged in academic research because it was a requirement for graduation. One of the program directors explained that “most of my residents are contributing to scientific research because it is a mandatory requirement for graduation by Saudi Commission for Health Specialties.”

For the third theme of trainer’s behaviors in educational program, the subtheme of resident lifestyle emerged because the program directors stated that residents taking care of themselves and the threat of burnout were important factors that influenced training behaviors. One of the program directors explained that the “amount of sleep and eating habits as examples of wellbeing are really important and create a healthy environment for the residents.” Another program director explained the problem of burnout among the residents: “the main negative factor that is really problematic to any residents is burnout.”

The second subtheme that emerged related to trainer’s behaviors in educational program was the ethics and professionalism of the residents. One of the program directors explained that residents must learn how to be welcoming to others:

“It’s different. Some of the residents are quiet and they are not welcoming to any new people, and some are welcoming. Part of those unwelcoming residents are forced to be welcoming and communicating well to patients and other staff as this is one of the learning outcomes in their residency program” (1-1-1-1).

Another program director explained the importance of residents building good relationships with other residents and with nurses:

“The extent of collaboration among all residents is strongly dependent on how good the relationship between each other and with nurses is. Moreover, building a good and fruitful relationship between each other needs time and that’s why the senior residents are more effective team members than PGY-2 residents” (2-1-1-2).

Overall, the results showed that the program directors indicated that the residents needed to improve their documentation skills. The program directors were also not satisfied with the manager role of the residents, particularly regarding healthcare resource utilization. In the scholar role, the program directors believed that the residents were conducting research not out of a personal desire to conduct research, but because it is a requirement for graduation. The majority of program directors believed that several influential factors that might affect the residents’ overall performance included stress and the receipt of constructive feedback.

Mixed methods

Based on the researcher’s subjectivity, four areas were measured namely, confidence level and age, confidence level and gender, confidence level and training hospital, and confidence level and training level in Table 14.

**Table 14:** Mixed Methods Methodology Results

Four areas measured	Quantitative	Qualitative	Mixed Methods
Confidence level and Age	No significant correlation between level of confidence and age	PDs indicate no changes were made	Similar
Confidence level and Gender	No significant correlation between level of confidence and gender	PDs indicate changes were made	Different
Confidence level and Training Hospital	No significant correlation between level of confidence and training hospital	PDs indicate no changes were made	Similar
Confidence level and Training Level	No significant correlation between level of confidence and training level	PDs indicate changes were made	Different

In the first area “confidence level and age”, quantitively there was no significant correlation between level of confidence and age, and qualitatively the program directors indicate no changes were made in this regard by saying for example “Leadership skills rely on the training and the residents themselves rather than the ages or genders. I see the senior residents are more leaders than junior residents.”

The second area measured “confidence level and gender”, quantitively there was no significant correlation between level of confidence and age, and qualitatively the program directors indicate changes were made in this regard by saying for example “Clinically, most of my residents especially those with same gender are feeling comfortable to work with each other and being more effective teamwork members.”.

The third area measured “confidence level and training hospital”, quantitively there was no significant correlation between level of confidence and age, and qualitatively the program directors indicate no changes were made in this regard by saying for example “I do believe that it is within the residents’ capacity to excellent procedures if they make it their priority and want to do more procedures.”

Lastly, the fourth area measured “confidence level and training level”, quantitively there was no significant correlation between level of confidence and training level, and qualitatively the program directors indicate changes were made in this regard by saying for example “The standard of care is different among my residents, and it depends on their experience and confidence as they grow from level to level.”

Therefore, both data sets were convergent between confidence level and all independent variables (resident’s age, gender, and training level) except the training hospital which was divergent.

## Discussion

This chapter discussed the findings of each research question, where justified and debated with referring to previous studies in either supporting or contracting current findings.

This mixed methods study was conducted based on the recommendation of recent evidence as it can enhance the applicability and integrity of findings [11,15]. It was aimed to assess the residents’ confidence level, and to measure the program directors’ satisfaction in the residents’ performance during their training years within the SBEM program in Riyadh hospitals. As of now, no study has addressed the direct effect of educators on their residents’ performance [2-5].

Quantitative method findings

In the current research study, the researcher studied 120 respondents who were residents that actively joined the SBEM program in all residency training hospitals in Riyadh City, Saudi Arabia. In order to verify the previous aim, descriptive (frequency, percentage, mean, and standard deviation) and inferential analysis (Fisher’s exact test) were used in this study. The results showed that, there was no statistically significant correlation between the level of confidence and respondent’s (gender - age - training level - training hospital).

The results of the quantitative findings allow for the ability to understand how confident the residents in this study were about the specific academic and clinical performance indicators that were examined. These results showed that the residents who were surveyed in this study had the highest level of confidence in acting as collaborators. Below confidence as collaborators, the residents had a slightly lower level of confidence acting as communicators and as professionals. However, the mean score for scholar was much lower than the other three clinical and academic performance indicators.

The conclusion that can be drawn from the lower score on the scholar dimension of performance as compared to collaborator, communicator, and professional is that the residents had much more difficulty perceiving themselves as scholars. It is important to note that with the data that were collected, it is not possible to understand whether the residents in this study had a lower perception of themselves as scholars because they had difficulty with research activities or because they did not want to act as scholars.

Qualitative method findings

In this research, the researcher studied nine program directors in Riyadh training hospitals. In order to verify the previous aim, thematic analysis was used in this study, and the results showed that:

The program directors were satisfied with all roles of CANMEDs framework among their residents except “Communicator, Manager, and Scholar” roles. Firstly, in the “Communicator” role, the program directors believed that the residents need to improve their skill of documenting medical records. Secondly, in the “Manager” role, all program directors were unsatisfied regarding healthcare resources utilization. Thirdly, in the “Scholar” role, program directors perceive that most of their residents are conducting research because it was a mandatory requirement for graduation from the Saudi Commission for Health Specialties. In addition, most program directors mentioned several influential factors that might affect the residents’ overall performance positively or negatively like, stress and receiving constructive feedback which have been mentioned in previous literature. One study concluded that emergency medicine residency program supervisors should be aware of the effects of stress on the residents that might have an impact on their performances [10]. Also, giving a well-constructed feedback will affect the resident’s performance [7]. Another study was conducted by Alsalamah and Almadani, which found that the SBEM residents in Riyadh city were generally satisfied with their program but the constructive feedback was insufficient and needed to improve [8].

An interesting finding from the interviews conducted with the program directors was that the program directors stated that the residents were only conducting research because it was a mandatory for graduation. This finding may help to explain why the residents had the lowest scores on the scholar aspect of the quantitative surveys. The residents may have had the lowest scores related to clinical and academic performance regarding their roles as scholars because they did not want to engage in research. Rather than having anything to do with confidence about their abilities, the quantitative scores related to the scholar role may have been about a lack of desire to engage in research on the part of the residents.

Mixed methods findings

Both quantitative and qualitative data sets were convergent between confidence level and all independent variables: resident’s age, gender, and training level, except the training hospital which was divergent. Although in the area of “Confidence level and gender” the result of mixed methods was different, we consider it convergent because some program directors said that “feeling comfortable” has been likened to feeling confident which is not the same thing (i.e., feeling comfortable with someone does not mean being more confident to work with that person).

Generally, confidence is expressed in different ways based on the circumstances and the response to different modifiers, it is important to consider both confidence and competence, as well as their relationship [15].

Given the importance of residents’ confidence and its relationship with their competency, as well as the possible influential factors that could affect the residents’ academic and clinical performance, the literature suggests that conducting practical sessions for instance, simulation sessions would help the residents to improve their self-confidence and therefore, to decrease the stress and anxiety levels which in turn could foster their competency as future emergency medicine physicians [16].

## Conclusions

The aim of this study was to assess the residents’ confidence level, and to measure the program directors’ satisfaction of the residents’ performance during their training years within the SBEM program in Riyadh hospitals. The importance of this study is indicative in the lack of previous research in this area. It is generally assumed that emergency medicine educators can have a great influence over the academic and clinical performance of residents. However, without a scientific study, the actual influence that emergency medicine educators have on residents is not known.

Based on our data analysis, we can conclude that residents in the SBEM residency program exhibited high confidence in their academic and clinical performance, primarily influenced by their institutional educators. Demographic factors such as age, gender, and training hospital did not significantly impact residents' confidence levels, except for their training level. Notably, it was the educators who played a pivotal role in shaping and influencing the residents' academic and clinical performance.

However, the results of the data analysis showed that the PDs thought there were some issues with the performance of the residents. Specifically, they indicated that the residents needed to improve their documentation skills and their healthcare resource utilization. Furthermore, they believed that the residents conducted research because it was a requirement for graduation. Most of the PDs also believed that several influential factors that may have affected the residents’ overall performance included stress and giving constructive feedback.

## Appendices

Research Questionnaire - Qualitative Study

Title: The Influence of Saudi Board of Emergency Medicine Residency Educators on Residents’ Academic and Clinical Performances in Riyadh, Saudi Arabia; Mixed Method Methodology

Dear program director, the aim of this survey is to explore your own perception toward your PGY-2 to 4 SBEM -residents academic and clinical performance as compared to other Riyadh training hospitals.

Overall, the perception is based on CANMEDs framework that include medical expert, communicator, collaborator, manager, scholar, health advocate, and professional, using In-Training Evaluation Report (ITER).

Kindly, answer as honestly and as objectively as you can. Please note that by answering the following questions you consent the authors to use the information provided for research purposes. Thank you in advance for your kind participation.

**Table 15:** Demographic Data

Demographic Data	
Gender	Male
	Female
Program director experience	< 1 year
	1 – 2 years
	3 – 4 years
	> 5 years
I supervise SBEM residents at	Prince Sultan Military Medical City
	King Abdulaziz Medical City
	King Fahd Medical City
	King Saud Medical City
	Prince Mohamed bin Abdulaziz Hospital
	Security Force Hospital
	King Faisal Specialized Hospital
	King Khalid University Hospital
	Dr. Sulaiman Alhabib Hospital

**Table 16:** Qualitative Questionnaire Items

No.	Question
1	Briefly, describe your professional background?
2	What do you think about the emergency approach of your residents?
3	Why do you think that the management strategies are varied from resident to resident in same level?
4	If you do agree with the ability variation of performing emergency procedures among the residents, Justify such a variation?
5	Explain the awareness of the role of evidence among the different levels of residents in your institution?
6	If you do agree that the communication skills with patients and HCPs among your residents is apparently different, clarify why such a difference is existing?
7	From your perspective, up to what extent the medical records are sufficiently written by your different levels of residents?
8	8. Describe how your different residents are being effective teamwork?
9	Justify why the leadership skills is performed differently among your residents?
10	Give your opinion regarding health care resources utilization by your residents?
11	Clarify up to what extent the residents are accepting and making changes to given feedback?
12	Tell me about how your residents are contributing to education of patients, junior staff?
13	Tell me about how your residents are contributing to scientific research?
14	If you do agree that the health advocacy with patients among your residents is varied, clarify why such a variation is existing?
15	What do you think about the standard of clinical care and seeking advice among different levels of residents in your institution?
16	Based on the previous answers, up to what degree do you level the impact of your institutional educators on the SBEM residents PGY-2 to 4?
17	From your perspective, what are the possible influential factors that might affect your residents academic and clinical performances (positively and negatively)?

Research Questionnaire - Quantitative Study

Title: The Influence of Saudi Board of Emergency Medicine Residency Educators on Residents’ Academic and Clinical Performances in Riyadh, Saudi Arabia; Mixed Method Methodology

Dear Participant, the aim of this survey is to assess your own confidence toward your academic and clinical performance that are induced by your institutional educators/EM consultants using In-Training Evaluation Report (ITER). Kindly, answer as honestly and as objectively as you can. Please note that by answering the following questions you consent the authors to use the information provided for research purposes. Thank you in advance for your kind participation.

**Table 17:** Demographic Data

Demographic Data	
Gender	Male
	Female
Age in years	25 – 29
	30 – 34
	35 – 39
	> 39
I am a Saudi board of emergency medicine resident	PGY – 2
	PGY – 3
	PGY – 4
My training hospital is	Prince Sultan Military Medical City
	King Abdulaziz Medical City
	King Fahd Medical City
	King Saud Medical City
	Prince Mohamed bin Abdulaziz Hospital
	Security Force Hospital
	King Faisal Specialized Hospital
	King Khalid University Hospital
	Dr. Sulaiman Alhabib Hospital

**Table 18:** Quantitative Questionnaire Items

No.	Item	Not confident at all	Slightly confident	Somewhat confident	Fairly confident	Completely confident
		1	2	3	4	5
A. MEDICAL EXPERT						
1	I am comprehensive, accurate and concise with all relevant details about the history and physical examination.					
2	I use the diagnostic tests in a cost-effective manner and understands limitations and predictive value.					
3	I am able to formulate an appropriate differential diagnosis.					
4	I am able to analyze, integrate, and formulate effective management strategies.					
5	I have a broad clinical and basic knowledge of a wide variety of medical problems and develops a plan of secondary prevention.					
6	I am able to identify and respond appropriately to urgent cases.					
7	I am aware of the role of evidence in clinical decision-making.					
8	I am able to apply relevant information in problem-solving.					
9	I am able to demonstrate knowledge of medications used, mechanisms of action, clinically relevant pharmacokinetics, indications, contraindications, and adverse effects.					
10	I am able to perform diagnostic and therapeutic procedures, understand indications, limitations and complications.					
B. COMMUNICATOR						
11	I am able to communicate effectively with patients, their families, and HCPs.					
12	I am able to maintain clear, accurate and appropriate records.					
13	I am able to write an organized and legible orders and progress notes.					
14	I am able to document a concise and complete discharge summaries promptly.					
C. COLLABORATOR						
15	I am able to work effectively in a team environment with attending, juniors and nursing staff.					
D. MANAGER						
16	I am able to serve in administration and leadership roles as appropriate.					
17	I am able to use the health care resources appropriately and efficiently.					
E. SCHOLAR						
18	I attend and contribute to rounds, seminars, and other learning events.					
19	I accept and act on constructive feedback.					
20	I contribute to the education of patients, junior residents, house staff, and students.					
21	I contribute in scientific research.					
F. HEALTH ADVOCATE						
22	I am able to identify the psychosocial, economic, environmental and biological factors which influence the health of patients and society.					
23	I offer advocacy on behalf of patients at practice and general population levels.					
G. PROFESSIONAL						
24	I deliver the highest quality of care with integrity and compassion, and I recognize limitations and seek advice and consultations when necessary.					
25	I reflect the highest standards of excellence in clinical care and ethical conduct. Strongly disagree					
